# Amyloid-like RIP1/RIP3 RHIM Fragments’ Characterization and Application as a Drug Depot

**DOI:** 10.3390/molecules28031480

**Published:** 2023-02-03

**Authors:** Maytham Ismail, Mathumai Kanapathipillai

**Affiliations:** Department of Mechanical Engineering, University of Michigan-Dearborn, Dearborn, MI 48128, USA

**Keywords:** aggregation, RIP1, RIP3, Amyloid

## Abstract

Amyloid aggregates play a major role in diseases as well as in normal physiological function. Receptor-interacting protein kinases 1 and 3 (RIP1/RIP3) aggregates complexes in cellular necroptosis is one example of protein aggregation in normal cellular function. Although recently there have been several studies on full kinase proteins aggregation, the aggregation potential of small peptide sequences of RIP1/RIP3, the physicochemical properties, and the potential in biomedical applications have not been explored. Hence, in this paper, we study the aggregation propensity of peptides consisting of four and twelve amino acid sequences in the RHIM region of RIP1/RIP3 proteins that are known to drive the beta-sheet formation and the subsequent aggregation. The aggregation kinetics, physicochemical characterization, mechanosensitive properties, cellular effects, and potential as a cancer drug depot have been investigated. The results show that the number and concentration of amino acids play a role in amyloid-like aggregates’ properties. Further, the aggregates when formulated with cisplatin-induced significant lung cancer cell toxicity compared to an equal amount of cisplatin with and without ultrasound. The study would serve as a platform for further investigation on RIP1/RIP3 peptide and protein aggregates, their role in multiple cellular functions and diseases, and their potential as drug depots.

## 1. Introduction

Amyloid aggregation is a major topic of interest in the fields of protein research and clinical medicine [1,2,3,4,5,6]. Protein aggregation transforms the normal form of a protein into the misfolded form, which involves a transition of alpha-helical structure elements into the beta sheet [7,8]. The characteristic property of amyloid, which is a condensation of fibrils, is that a monomer can polymerize into an oligomer, and an oligomer can polymerize into fibrils. Protein misfolding is a result of both normal protein function and the cellular environment and can exist naturally or pathologically. The amyloid aggregates under pathological conditions cause ROS (reactive oxygen species) production and toxicity leading to disease onset [9,10,11]. Neurodegenerative disorders such as Alzheimer’s disease result from the pathologic aggregate of the amyloid protein [12,13]. Normal amyloid protein aggregation includes complexes of RIP1/3, RIP3, and RIP3 during necroptosis [14].

Although many studies have been reported on disease-causing protein aggregation, not many studies have been conducted on the protein aggregates that play a role in normal physiological functions. Previously, protein aggregation has been viewed as a pathological process due to its role in various neurological and aging diseases. Recently, many protein aggregates that contribute to normal cellular process have been identified [15]. Beneficial protein aggregates have shown to play key roles in physiological functions including the following: promotion of necrosis, support of memory, antimicrobial responses, storage and scaffolding [2,4,5,6,16,17,18]. Further, recently, there is interest in fabricating amyloid-like functional materials [19]. The intrinsic bioactive and biocompatible nature of protein and peptide-based materials make them an attractive alternative to conventional functional materials. Amyloid structures are highly organized structures with a few nanometers in diameter and a few micrometers in length. The structural and chemical properties of the amyloid enable their use in various biomedical applications such as scaffolds, drug depots, protein/drug packaging. If a beneficial amyloid-like aggregate that will not be detrimental to normal cells is discovered, it could be used for various therapeutic applications.

Among the protein aggregation processes relevant to the normal physiological process, the amyloid complex formed by receptor-interacting protein (RIP) kinases is one of the key examples [14,20,21]. It has been reported that RIP1/RIP3 proteins form an amyloid-like signaling complex that is essential for the necroptosis process which plays a key role in the normal physiological process [14,20,22]. The family of receptor-interacting protein kinases contain seven members; all have a kinase domain (KD) and are important regulators of cell death and survival [23,24]. Among the RIP kinases, receptor-interacting protein kinase 1/3 play a major role as signaling molecules in necroptosis, which is programmed necrosis [20,25]. The RIP kinases proteins have unique C-terminal motifs. RIP1 contains a C-terminal death domain, where RIP1 can engage in signal complexes that initiate different pathways [26,27]. RIP1 acts as a main switch of cell destiny regulation. By controlling the activation of pleiotropic cytokine tumor necrosis factor alpha (TNF-alpha), RIP1 plays a role in NF-kB activation, apoptosis, and programmed necrosis [28]. RIP1 receptor-interacting serine/threonine kinase1 was firstly identified in 1995 [29]. The RIP 1 protein has 671 amino acids. It contains a N-terminal serine/threonine kinase domain (KD) of 1 to 289 amino acids and an intermediate domain (ID) (290–582 amino acids) as well as a death domain (DD) (583 to 671 amino acids). The kinase domain has varied function in cell survival, and it is important for necroptosis induction. The intermediate domain contains the RIP homotypic interaction motif (RHIM) (531 to 547 amino acids) [20], which enables the protein to combine with RIP3. The assembly of the pro-necrotic RIP1–RIP3 complex is mediated through the RHIM region [28]. RIP3 receptor-interacting serine/threonine kinase 3 was discovered in 1997 [30]. The RIP3 protein has 518 amino acids. It contains a N-terminal serine/threonine kinase domain (KD) (1 to 287 amino acids), an intermediate domain (ID) (287 to 440 amino acids), and a unique C-terminal without a death domain (DD) (441 to 518 amino acids). The C-terminal domain contains a RIP homotypic interaction motif (RHIM) (440 to 451 amino acids) and mediates RIP3 interaction with RIP1 [20]. The C-terminus of RIP3 contains the RIP homotypic interaction motif (RHIM), whereas a RIP homotypic interaction motif (RHIM) is found in the intermediate domain of RIP1 [31,32]. During necroptosis, the RHIM domain is required for the interaction between RIP3 and RIP1 and likely mediates protein–protein interactions [33]. Through these interactions, the kinases have shown to form a functional amyloid structure that is essential for the necrosis process [22,33]. 

Recently, studies have been reported on the amyloid formation of RIP1/RIP3, where it has been shown that the RHIM (RIP homotypic interaction motifs) of the RIP1 and RIP3 proteins are responsible for the amyloid-like filamentous structure [6,7]. Further studies reveal that the core region containing the sequences IQIG and VQVG of the RIP1 and RIP3 proteins’ RHIM regions, respectively, is the key driver of the double beta sheet structure via the Ile and Val amino acid residues [6,7,34]. Studies have shown that the RIP1 residues (496–583) and RIP3 residues (388–518) in the RHIM region are able to form the amyloid structure formation [14,35,36]. To date, studies have reported the role of the full RIP1/RIP3 protein and the full RHIM region peptide aggregates. Investigations on short RHIM peptide sequences and their potential application have not been conducted yet. Regarding the RIP1 and RIP3 core region sequences of IQIG and VQVG, respectively, it is assumed that the Ile and Val amino acid residues form the double beta sheet structure, similar to the VQIVYK sequence of tau protein, which is responsible for aggregation [14]. Hence, short peptide sequences of the RIP1 and RIP3 proteins that would form amyloid-like aggregates would be an ideal candidate for aggregation studies, due to their relatively easy handling and cost compared to the full-length proteins. Further, there have been no studies yet on the potential therapeutic applications of RIP1/3 protein aggregates as drug depots. Due to their intrinsic therapeutic qualities, adaptability, biodegradability, and biocompatibility, protein aggregates offer beneficial properties over conventional nanocarriers. Therefore, RIP1/RIP3 aggregates could be used as drug delivery vesicles, and this is a further motivation of this study.

In this manuscript, we have investigated whether short amino acid sequences in the RHIM region would be able to exhibit amyloid structural properties and potential functional properties. Further, short amino acid sequences consisting of the core region of the RIP1 and RIP3 proteins that would form aggregates and would exhibit functional properties would have added benefits due to the relatively easier handling and lower cost. Here we report the aggregation characteristics of two amino acid sequences, each comprising 4 amino acids (IQIG, VQVG) and 12 amino acids (TIYNSTGIQIGA, NIYNCSGVQVGD) of the RIP1 and RIP3 proteins in the aggregation prone core region. Further, compared to conventional nanocarriers, the use of amyloid-like structures as therapeutic drug depots has not been explored in detail. Protein/peptide/amyloid aggregates with inherent physiological properties could have added advantages as drug depots compared to existing drug delivery depots. Cisplatin is used as a model cancer drug, and the effect of the peptide aggregate/cisplatin formulation in lung cancer cells is studied. The study reported would serve as a platform for RIP1/RIP3 amyloid complex-based biomedical applications. 

## 2. Results

**Figure 1:**
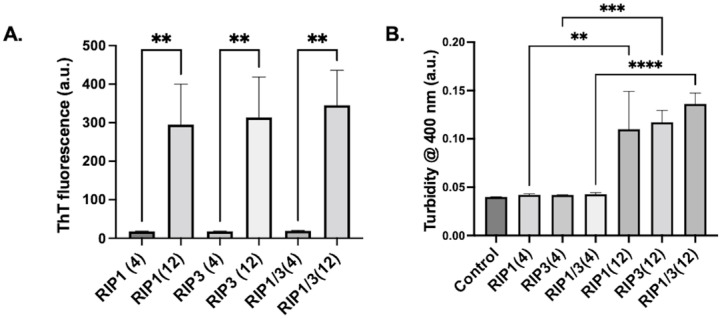
(**A**) Thioflavin-T characterization of aggregation of RHIM fragments of RIP1, RIP3, and RIP1/RIP3 complex after 1 day. RHIM fragments composed of 4 amino acid residues (RIP1(4), RIP3(4), RIP1(4)/RIP3(4)) and 12 amino acid residues (RIP1(12), RIP3(12), RIP1(12)/RIP3(12)) were used for the study. (**B**) Turbidity measurement of the corresponding RHIM fragment aggregates after 1 day. n = 3 ± SEM, ** *p* < 0.01, *** *p* < 0.001, **** *p* < 0.0001.

First, we tested the amyloid-like potential of 4 amino acid sequences and 12 amino acid sequences of RIP1 and RIP3 proteins. Three different peptide conditions, RIP1, RIP3, or RIP1/RIP3 (combination of RIP1 and RIP3), were used for the study. Peptide concentrations of 1 mM was aggregated in 100 mM Ammonium Acetate, pH 7, and the aggregation kinetics were measured at 1 day. Thioflavin T (ThT) and turbidity measurements were performed to assess the aggregation. The short peptide sequence that comprises the aggregation prone core RHIM region did not exhibit significant aggregation, as can be seen from thioflavin T and turbidity measurements shown in Figure 1. On the other hand, the 12 amino acid peptides comprising the expanded core RHIM region exhibited significant aggregation, as indicated by the ThT data shown in Figure 1A. Similar results were observed with turbidity measurements (Figure 1B). Thus, numbers of amino acids in the RHIM fragment remarkably affect the aggregation. 

**Figure 2:**
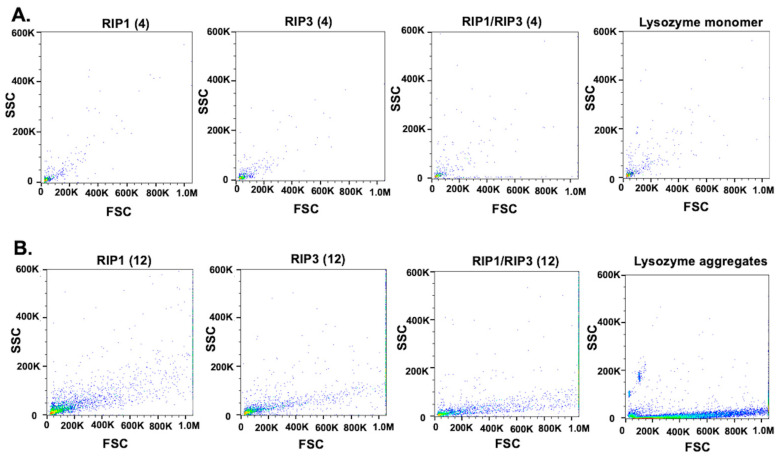
Peptide aggregation detection with proteostat assay. RHIM fragments were aggregated for 1 day in 100 mM ammonium acetate buffer, pH 7. Lysozyme monomers and aggregates were used as controls. (**A**) Four amino acid fragments exhibited a monomers-like scattering profile similar to lysozyme monomers. (**B**) Twelve amino acid fragments exhibited a protein aggregates-like scattering profile similar to the lysozyme aggregates.

To further confirm this, we performed proteostat assay, a commercial protein aggregation assay similar, in principle, to thioflavin T binding. We compared the peptide aggregates and monomers with the known lysozyme aggregates and monomers. The short peptides exhibited a monomers-like profile indicating no significant aggregation (Figure 2A). The long peptides showed an aggregates-like scattering profile (Figure 2B). The proteostat data are in alignment with the thioflavin T and turbidity data. 

**Figure 3:**
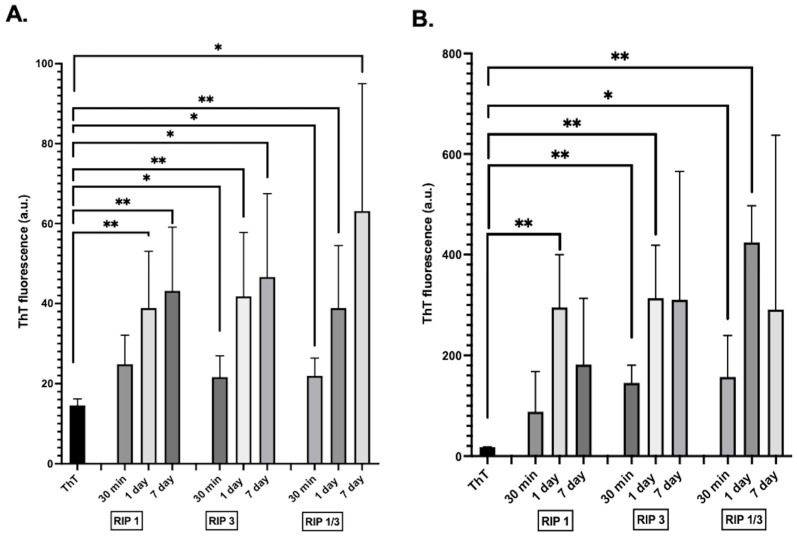
Effect of concentration on 12 amino acid RHIM fragments RIP1, RIP3, and RIP1/RIP3 aggregation. (**A**). ThT fluorescence of 100 µM peptides aggregation. (**B**) ThT fluorescence of 1 mM peptides aggregation. n = 3 ± SEM, * *p* < 0.05, ** *p* < 0.01.

Since the four amino acid peptide sequence did not exhibit significant aggregation under the tested conditions, we then focused our studies on the 12 amino acid sequences. As a next step we studied the effect of concentration and time on the 12 amino acids (TIYNSTGIQIGA, NIYNCSGVQVGD) of the RIP1 and RIP3 peptides aggregation. Both 100 µM and 1 mM concentrations exhibited significance aggregation (Figure 3). However, compared to 100 µM, 1 mM peptides showed an increase in ThT aggregation binding, indicating concentration-dependent aggregation. In addition, the aggregation kinetics reveal a growth of aggregation followed by a possible degradation by 1 week. 

**Figure 4:**
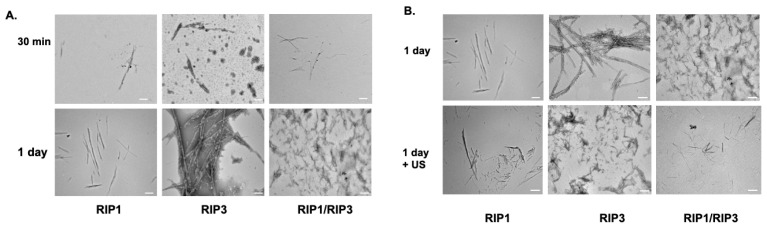
(**A**) Transmission electron microscopy (TEM) images of 12 amino acid RHIM fragments RIP1, RIP3, and RIP1/RIP3 aggregation kinetics. The images reveal significant aggregation after 1 day compared to 30 min. Scale bar 100 nm. (**B**) Ultrasound (US) mediated mechanosensitive effects of aggregates assessed with TEM. The images reveal the aggregates mechano sensitivity to ultrasound 2.2 W/cm^2^, 5 min. Scale bar 100 nm.

**Figure 5:**
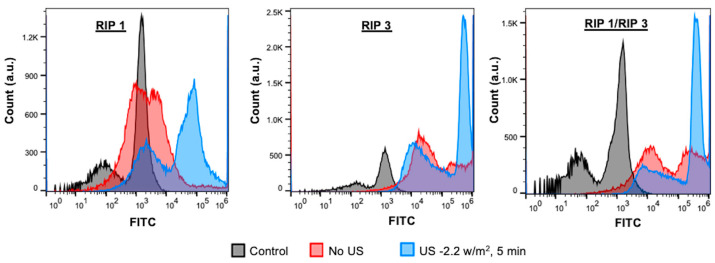
Ultrasound mediated uptake of the aggregates assessed with flowcytometry. The scattering measurements reveal cellular uptake of RIP1, RIP3, and RIP1/RIP3 aggregates were increased in the presence of ultrasound 2.2 W/cm^2^, 5 min.

Next, TEM was used to determine the morphology of the aggregates. TEM images of 1 mM peptides exhibited a fibrous-like structure one day after aggregation, indicating amyloid-like behavior (Figure 4A). We then tested whether the amyloid structures have mechanosensitive properties. The amyloid structure’s stability was tested in the presence of ultrasound. Peptide aggregates were subjected to ultrasound intensity 2.2 W/cm^2^ for 5 min and imaged with TEM (Figure 4B). The structures disintegrated into smaller aggregates and fibers indicating mechanosensitive properties. Next, we tested whether the cells were able to uptake the aggregates with or without ultrasound. As can be seen from the flowcytometry measurements, there is an enhanced fluorescent count; hence, the increase in uptake was seen in the presence of ultrasound treatment (Figure 5). 

**Figure 6:**
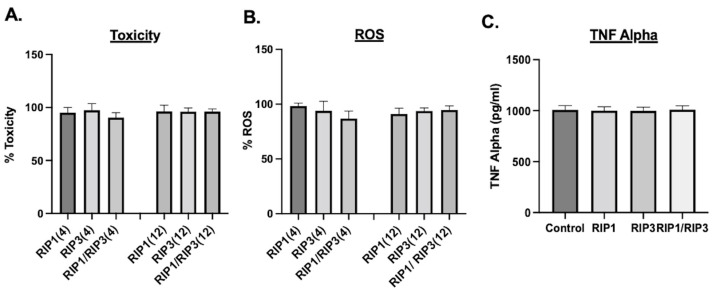
(**A**,**B**) Effect of aggregates on normal cell toxicity and ROS. Normal lung BEAS-2B cells were plated in 96 wells at a density of 10,000 cells/well. RIP1/RIP3 aggregates were added at a concentration of 10 µM and incubated for 48 h, and Alamar blue/ROS measurements were performed. (**C**) TNF alpha assay. Raw264.7 cells were plated at a density of 10,000 cells/well in 96 well plates. RIP1/RIP3 aggregates were added at a concentration of 100 µM and incubated for 48 h. TNF-alpha assay was performed according to the manufacturers protocol, and measurements were obtained using a M3 plate reader.

Next, we tested whether the amyloid structures induce toxicity, reactive oxygen species (ROS), or an immune response to normal cells. BEAS-2B normal lung cells were used for the toxicity and ROS study. As can be seen from Figure 6A, no significant toxicity was observed with 10 µM of peptide aggregate treatment. Since amyloid aggregates are known to induce oxidative stress, we performed ROS measurements. ROS experiments were performed in a similar set up; after 48 h of the aggregate’s treatment, cells were treated with H2DCFDA for 30 min, and the ROS effects were measured. No significant oxidative stress was observed in BEAS-2B cells (Figure 6B). In addition to the toxicity and ROS study, we also performed tumor necrosis factor α (TNF-α) immune response assay as amyloid aggregates are known to induce macrophage activation [37,38]. Raw 264.7 macrophage cells were treated with 100 µM of the peptide aggregates, and TNF-α response was measured. As shown in the Figure 6C, no significant immune response was exhibited by the aggregates. Therefore, the peptide aggregates could be used for biomedical applications without any side effects. 

**Figure 7:**
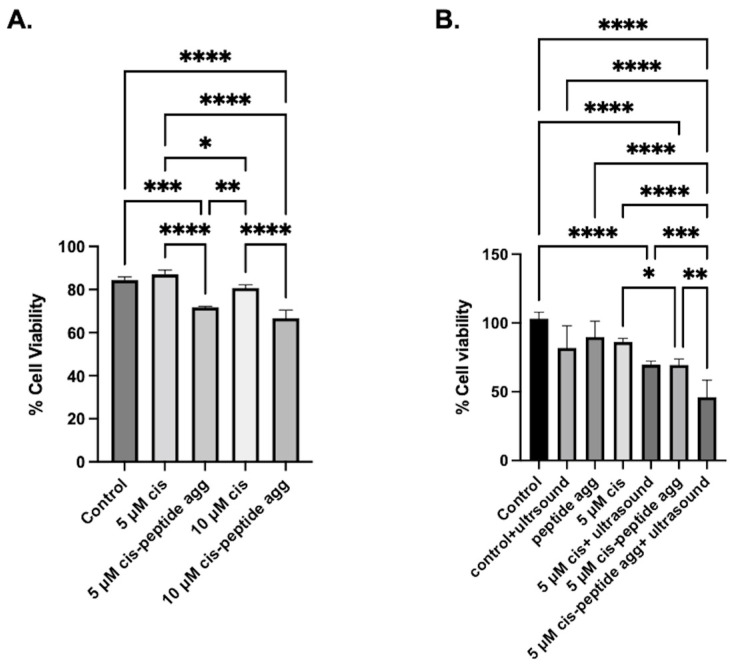
(**A**) LDH toxicity assay of H1299 cells. Cells were treated with 5 µM and 10µM cisplatin alone or encapsulated with RIP1/RIP3 peptide aggregates. Supernatant from the treated cells were treated with LDH reagents, and the absorbance measurements were taken at 490 nm. Control consisted of lysate from control cells in the absence of peptide aggregates or cisplatin. (**B**) Ultrasound-mediated delivery of RIP1/RIP3 peptide aggregates encapsulated with cisplatin significantly improve cellular toxicity. LDH toxicity assay was of H1299 cells treated with cisplatin conditions with and without ultrasound 1.6 W/cm^2^, for 3 min. Controls consisted of lysate from control cells in the absence of peptide aggregates or cisplatin with and without ultrasound. n = 3 ± SEM, * *p* < 0.05, ** *p* < 0.01, *** *p* < 0.001, **** *p* < 0.0001.

Finally, we tested the potential of the amyloids as drug delivery depots. Cisplatin was chosen as a model drug to treat H1299 lung cancer cells. Cisplatin was encapsulated within peptides aggregates, and the encapsulation efficiency was found with o-phenylenediamine (OPD) assay. The cisplatin calibration curve was obtained at the 1.5–15 µg/mL range (corresponding to 10–100% of cisplatin used for encapsulation), and the amount of cisplatin in the peptide aggregates was calculated at 705 nm. The amount of cisplatin encapsulated was found to be 7.71 ± 0.20 µg/mL. For the study, either 5 or 10 µM of cisplatin-encapsulated aggregates was used. As a control, peptide aggregates formulated under similar conditions without cisplatin were used. LDH cytotoxicity assay was performed to assess the toxicity effects of cisplatin and cisplatin-encapsulated peptide aggregates. Results show that cisplatin-encapsulated aggregates show significant toxicity compared to the same amount of the cisplatin alone treatment (Figure 7A). The cell viability was observed to be around 63% and 70% with peptide aggregates encapsulated with cisplatin with a concentration of 5 or 10 µM. On the other hand, for the cisplatin-alone-treated cells, the viability was above 80%. Next, the efficacy of the formulation was tested in the presence of ultrasound 1.6 W/m^2^, 3 min. The ultrasound power and time were chosen to minimize the toxicity effects due to ultrasound alone. The ultrasound mediated delivery further enhances the efficacy, in that the cell viability was reduced to around 40% as can be seen in Figure 7B. The results indicate the potential of the aggregates as drug delivery vesicles. 

## 3. Discussion

In this paper we have shown that the RIP1/RIP3 peptides have amyloid-like properties and also have the potential to be used as drug delivery vesicles. The study reveals that the short amino acid peptides RIP1(IQIG) and RIP3 (VQVG) did not exhibit significant aggregation. On the other hand, peptides comprising 12 amino acids, RIP1 (TIYNSTGIQIGA) and RIP3 (NIYNCSGVQVGD), exhibited significant aggregation properties at concentrations above 100 μM. The results suggest that the longer the aggregation-prone RHIM peptide sequence, the easier it is to nucleate and form aggregates in vitro. The smaller the peptide sequence, the higher the concentration needed to form aggregates. Further, the results show that the aggregates’ size would be tuned by ultrasound mediated breakage of the aggregates. To achieve narrow distribution of the amyloid aggregates, further optimization of the aggregation conditions should be performed in future studies as well as complementary size measurements such as dynamic light scattering, or atomic force microscopy could be utilized. Further, although OPD measurement is a widely accepted method for cisplatin detection, we found that OPD cisplatin complex is not very stable, and the absorbance values were sensitive to time and temperature. In the future, for cisplatin detection, a fluorescently labelled cisplatin could be used as an additional quantification method. Regarding the aggregate’s effects on cancer cells, the peptide aggregates-encapsulated cisplatin induces cytotoxicity to lung cancer cells, whereas equivalent free cisplatin did not exhibit any toxicity, and this shows the potential of peptide aggregates as drug depots. 

This is the first time that aggregation of short peptide sequences of RIP1/RIP3 has been studied in detail. Further applications of the peptide aggregates as drug depots were not explored before. Previous studies were either focused on full protein or peptides with a large number of amino acids covering the RHIM region [1,2,3,4]. Although peptide aggregation of RIP1/RIP3 has advantages compared to full length protein aggregation studies due to easier handling and lower cost, the functional effects of the aggregates may be limited. RIP1/RIP3 peptide sequence only contain the aggregation-prone region but not the kinase domain of the protein, which is essential for the necroptosis function of the protein. Another limitation with peptide aggregates is the need for a rather high concentration to form aggregation. 

Hence, future studies should focus on the investigation of both the full protein aggregation and small peptide sequences to have a clear understanding about the mechanism of RIP1 and RIP3 amyloid aggregates, as well as structural and functional properties, to realize their potential in biomedical applications. Beneficial protein/peptide aggregates are formed under normal physiological conditions and, therefore, have different aggregation formation mechanisms. Hence, detailed characterization studies need to be performed to understand the mechanisms of aggregation. Due to the inherent therapeutic properties, peptide/protein aggregates have added advantage as drug carriers compared to conventional formulations; however, to realize successful therapeutic depots, the amyloid aggregates’ size and morphology must be tightly controlled. Further, aggregation conditions need to be tuned to achieve novel structural and functional properties. In summary, the study paves a way for further research on investigating RIP1 and RIP3 peptides and proteins’ aggregation in vitro and in vivo, and invites additional studies on their potential applications in treating diseases.

## 4. Materials and Methods

### 4.1. Materials

Receptor interacting protein RIP 1 and RIP 3 peptides were custom synthesized from Genscript (Piscataway, NJ, USA). The other materials and reagents were bought from either Thermo Fisher Scientific (Waltham, MA, USA) or Sigma Aldrich (St. Louis, MO, USA).

### 4.2. Aggregation Formation

Peptide aggregation was performed using two peptide sequences, IQIG, TIYNSTGIQIGA, VQVG, NIYNCSGVQVGD of RIP1 and RIP3, respectively. First peptide stock solutions in DMSO at 10 mM, 50 mM, and 100 mM concentrations were prepared. From the stock solutions, 20 μM, 100 μM, or 1 mM concentrations of peptides were prepared by dissolving in 100 mM Ammonium Acetate, pH 7. Ammonium acetate was widely used as a buffer for amyloid peptides’ aggregation [39,40]. Hence, we have chosen ammonium acetate as the buffer for our studies. The peptide solutions were prepared at cold conditions to avoid spontaneous aggregation. The peptides were then aggregated at around 37 °C in a thermomixer in the lab. For detection in cellular assays, peptide aggregates were performed with FITC-labelled peptides. For drug depot study, peptides were mixed with cisplatin and aggregated for 1 h. The aggregates were then purified through centrifugal filtration to remove unencapsulated cisplatin. The amount of encapsulated cisplatin was determined with o-phenylenediamine (OPD) assay as previously reported [41,42]. Then, 1 mM cisplatin was mixed in 1 mM peptide solution and was aggregated for 1 h. Next, the samples were purified with centrifugal filtration (Amicon centrifugal filters, 3400 MWCO, Millipore Sigma, St. Louis, MO, USA) to remove free cisplatin. Cisplatin calibration curve was obtained at 1.5–15 μg/mL range (corresponding to 10–100% of cisplatin used for encapsulation), and the amount of cisplatin in the peptide aggregate was calculated at 705 nm. Cisplatin and o-phenylenediamine when reacted at 100 °C for 30 min forms a green color complex with maximum absorbance at 705 nm. Next, the peptide aggregation process was characterized through Thioflavin-T (ThT), proteostat assay, Turbidity, and Transmission electron microscopy (TEM) measurements as described below.

### 4.3. ThT Fluorescence and Proteostat Assay

Thioflavin T (ThT) has been shown to exhibit fluorescence properties upon binding to amyloid structures and, hence, is used as a reliable protein aggregation characterization technique [43,44]. Peptide aggregates were mixed with 1 mM ThT (50 μM, tris buffer, pH 8) to result in a final peptide aggregate concentration of 5 or 50 μM. The aggregation kinetics were measured at 30 min, 1 day, and 7 days at 440/482 excitation and emission wavelengths. The measurement was carried out using a spectraMax M3 spectrophotometer from molecular devices, San Jose, CA, USA. The experiments were repeated four times. For the proteostat assay (Enzo Life Sciences, Ann Arbor, MI, USA), 5% of aggregated peptides or monomers are used and compared with lysozyme standards according to the manufacturer’s protocol. Proteostat assay has been widely used as a characterization method to detect protein aggregation [45,46]. Measurements were carried out using an Attune NxT flow cytometer in the lab. 

### 4.4. Turbidity

Turbidity has been widely used as a characterization method to monitor protein aggregation [47]. Turbidity measurements were performed at 30 min, 1 day, and 7 days during the aggregation process. Turbidity was measured at 400 nm wavelength, using a spectraMax M3 spectrophotometer (San Jose, CA, USA). Experiments were repeated four times.

### 4.5. Transmission Electron Microscopy (TEM)

TEM was used to characterize morphological features of the aggregates. First, 200 mesh copper grids with carbon-coated formvar film were plasma-treated to render hydrophilic properties. Then 5 μL of the samples were spotted onto the grids and incubated for 5 min. Excess liquid was blotted out and then stained with 2% phosphotungstic acid pH 7.4 for 2 min. For the imaging, aggregates were collected at 0 h and 1 day during the aggregation process. Imaging was performed using a JEOL TEM microscope (Peabody, MA, USA) at 80 kV at the electron microscopy facility at the University of Michigan Ann Arbor medical school, USA.

### 4.6. Mechanical Characterization

To test whether the peptide aggregates exhibit mechano sensitive properties, ultrasound mediated mechano sensitive measurement was performed. Ultrasound was performed using ultrasonicator 740 from Mettler Electronic Corp, Anaheim, CA, USA (intensity 1.6–2.2 W/cm^2^, 1 cm^2^ applicator) in the laboratory. The sonication was applied to the aggregate solutions for 3, 5, or 10 min. The effect of ultrasonication on the aggregates was measured with TEM imaging and flow cytometry.

### 4.7. Cell Toxicity (alamarBlue) and ROS Assay

To assess whether the peptide aggregates induce any adverse effects to normal cells, alamarBlue assay and ROS assay were performed. BEAS-2B cells were obtained from ATCC (American Type Culture Collection) and cultured in RPMI, 10% fetal bovine serum and 1% antibiotic-antimycotic according to the protocol. Cells were cultured in a 96-wells plate at a density of 10,000 cells/well. Then after 24 h, cells were treated with 10 μM and or 100 μM of RIP 1, RIP 3 peptide aggregates and subsequently incubated for additional 48 h. For the toxicity assessment, alamarBlue assay was performed according to the manufacturer’s protocol. The cell toxicity was assessed through the metabolic activity of the cells at 570/590 nm, excitation/emission. For the reactive oxidative stress (ROS) detection, H2DCFDA assay (Thermo Fisher scientific, Waltham, MA, USA) was used as reported in studies [48,49]. DCFH-DA assay fluorescence was performed at 495/527 nm excitation and emission. Experiments were repeated four times.

### 4.8. TNF Alpha Assay

To test whether the peptide aggregates induce any immune response, TNF alpha assay was performed. Murine macrophage Raw264.7 cells were cultured in 96 wells at 20, 000 cells/well. After 24 h 10 μM and or 100 μM RIP1, RIP3 peptides aggregates were added to the cells and then incubated for additional 48 h. Immune response of the aggregates was tested using TNF alpha assay from R&D systems according to the manufacturer’s protocol. 

### 4.9. LDH Toxicity Assay

To measure the cisplatin-induced cellular toxicity, CyQUANT™ LDH Cytotoxicity Assay (Invitrogen, Waltham, MA, USA) was performed. H1299 lung cancer cells were cultured in RPMI, 10% fetal bovine serum and 1% antibiotic-antimycotic according to the protocol. For the toxicity assay, cells were cultured in a 96-wells plate at a density of 10,000 cells/well. Then after 24 h, cells were treated with 5 or 10 μM of cisplatin alone or RIP1/RIP3 peptide aggregates encapsulated with 5 or 10 μM of cisplatin with and without ultrasound (1.6 W/cm^2^, for 3 min) and subsequently incubated for 48 h, and LDH assay was performed according to the manufacturer’s protocol. The amount of LDH released was measured through the reduction of a tetrazolium salt to a red formazan product that was measured spectrophotometrically at 490 nm [50]. The level of formazan formation is directly proportional to the amount of LDH released into the medium, measuring cytotoxicity. Experiments were repeated four times.

### 4.10. Statistical Analysis

All experiments for the study were repeated at least three or more times. The data are represented as mean standard error (S.E.). Prism software (version 9.20), and Microsoft Excel (version 16.69) were used for the data analysis. Analysis of variance (ANOVA) followed by unpaired student *t*-tests or Tukey’s HSD post hoc analysis was used to determine the statistical significance. Data with significance are represented as * *p* < 0.05, or ** *p* < 0.01, *** *p* < 0.001, **** *p* < 0.0001.

## Data Availability

Not applicable.
